# Towards molecular evolutionary epigenomics with an expanded nucleotide code involving methylated bases

**DOI:** 10.1093/dnares/dsaf025

**Published:** 2025-09-16

**Authors:** Shinya Yoshida, Ikuo Uchiyama, Masaki Fukuyo, Mototsugu Kato, Desirazu N Rao, Mutsuko Konno, Shin-ichi Fujiwara, Takeshi Azuma, Ichizo Kobayashi, Hirohisa Kishino

**Affiliations:** Graduate School of Agricultural and Life Sciences, The University of Tokyo, Bunkyo-ku, Tokyo 113-8657, Japan; Laboratory of Genome Informatics, National Institute for Basic Biology, Okazaki 444-8585, Japan; Department of Molecular Oncology, Graduate School of Medicine, Chiba University, Chiba 260-8670, Japan; Department of Gastroenterology, National Hospital Organization Hakodate Hospital, Hakodate, Hokkaido 041-8512, Japan; Department of Microbiology and Cell Biology, Indian Institute of Science, Bangalore 560-012, India; Department of Pediatrics, Sapporo Kosei General Hospital, Sapporo, Hokkaido 060-0033, Japan; Department of Pediatrics, Sapporo Kosei General Hospital, Sapporo, Hokkaido 060-0033, Japan; Division of Gastroenterology, Department of Internal Medicine, Kobe University Graduate School of Medicine, Kobe 650-0017, Japan; Laboratory of Genome Informatics, National Institute for Basic Biology, Okazaki 444-8585, Japan; Department of Infectious Diseases, Kyorin University School of Medicine, Mitaka, Tokyo 181-8611, Japan; Research Institute for Micro-Nano Technology, Hosei University, Koganei, Tokyo 184-0003, Japan; University of Paris-Saclay, 91190 Gif-sur-Yvette, France; Graduate School of Agricultural and Life Sciences, The University of Tokyo, Bunkyo-ku, Tokyo 113-8657, Japan; Research and Development Initiative, Chuo University, Bunkyo-ku, Tokyo 112-8551, Japan

**Keywords:** epigenome evolution, DNA methylation, *Helicobacter pylori*, substitution rate

## Abstract

In molecular evolution analyses, genomic DNA sequence information is usually represented in the form of 4 bases (ATGC). However, research since the turn of the century has revealed the importance of epigenetic genome modifications, such as DNA base methylation, which can now be decoded using advanced sequence technologies. Here we provide an integrated framework for analyzing molecular evolution of nucleotide substitution, methylation, and demethylation using an expanded nucleotide code that incorporates different types of methylated bases. As a first attempt, we analysed substitution rates between bases, both unmethylated and methylated ones. As the model methylomes, we chose those of *Helicobacter pylori*, a unicellular bacterium with the largest known repertoire of sequence-specific DNA methyltransferases. We found that the demethylation rates are remarkably high while the methylation rates are comparable with the substitution rates between unmethylated bases. We found that the ribosomal proteins known for sequence conservation showed high methylation and demethylation frequencies, whereas the genes for DNA methyltransferases themselves showed low methylation and demethylation frequencies compared to base substitution. This study represents the first step toward molecular evolutionary epigenomics, which, we expect, would contribute to understanding epigenome evolution.

## 1. Introduction

The theory of molecular evolution, developed in the latter half of the 20th century, has laid a foundation for modern molecular biology by underpinning many of the tools used in genome analysis. In parallel with the explosion of genome sequence data around the turn of the century, our understanding of epigenetic modifications of DNAs, such as DNA base methylation, expanded. Now the entire genome sequences with methylated bases, the methylomes, can be decoded by the third generation sequencers.^1,2^

In eukaryotes, DNA methylation primarily occurs at the 5-position carbon atom of cytosine (5-methylcytosine; m5C). The m5C methylation is associated with transcription regulation across a wide range of eukaryotes although the patterns and mechanisms of methylation vary.^3^ In mammalian genomes, most m5C modifications are found at CG (CpG) dinucleotides. The abundance of CpG sites is much lower than expected because spontaneous deamination of m5C leads to a C-to-T transition mutation.^4^ Nonetheless, CpG methylation plays a crucial role in gene regulation and is involved in various biological processes.^5^ Increasing evidence suggests that the epigenomes of both animals and plants can be modified in response to environmental changes, influencing cellular gene expression and other biological processes.^6^

In prokaryotes, methylation also frequently occurs at the N6-position of adenine (N6-methyladenine; m6A) and the N4-position of cytosine (N4-methylcytosine; m4C) while these are rare in eukaryotes. Prokaryotic genomes typically carry a unique set of DNA methyltransferases, each recognizing a specific sequence motif. Many of these methyltransferases are paired with a restriction endonuclease to form a restriction–modification (R–M) system, which serves as a defense mechanism and a regulator of the expression of multiple genes. The other methyltransferases lack a partner restriction enzyme and play specific roles in gene expression regulation and other cellular processes.^7–9^ These enzymes transfer a methyl group to unmethylated double-stranded (ds) DNA as well as to hemimethylated ds DNA generated by DNA replication, converting it to fully methylated DNA and enabling the inheritance of the methylome.

In many multicellular eukaryotes, asexual reproduction allows the inheritance of the methylome, but their meiosis leads to reprogramming of most of the methylome. When epigenetic traits are passed to the next generation, a phenomenon known as *trans*-*generational epigenetic inheritance,* they have the potential to influence evolutionary processes. While trans-generational epigenetic inheritance is known to occur in plants, the extent to which it occurs in mammals remains a subject of ongoing debate.^10^

Cross-species comparative methylome analyses have previously been conducted among closely related eukaryotic organisms. A common approach is to compare methylation levels at CpG sites (or other sites in plants) within promoters or gene bodies of orthologous genes across the methylomes in equivalent tissues.^11–13^ Among other approaches, several have analysed evolutionary changes in methylation states at individual CpG sites under specific evolutionary models. For instance, phylogenetic relationships were inferred from methylome data based on a simple two-state model (methylated/unmethylated)^14^ or a multi-state model with discretized methylation rates.^15^ The evolutionary expansion of hypomethylated regions among mammalian germline methylomes was analysed using a phylo-epigenetic model, which enables the inference of ancestral states while accounting for the correlation of states at neighboring CpG sites.^16^ In plants, the rate of stochastic changes in methylation status (epimutation) has been evaluated using specifically maintained mutation-accumulation lines.^17,18^

Many bacterial species are unicellular and propagate through binary division, meaning that their germ line is equivalent to their somatic line. This characteristic, along with many advantages stemming from their simplicity, makes these bacteria excellent models for studying methylome evolution. However, bacterial methylomes are shaped by a variety of DNA methyltransferases that produce various types of methylated bases (m5C, m4C, m6A) on different sequence motifs, which adds complexity to their analysis.

In molecular evolution, evolutionary changes between multiple states, such as nucleotide substitutions, are modelled using a rate matrix. Various models of nucleotide substitution are available,^19–22^ many of which assume reversibility in the substitution process for mathematical simplicity. Among these, the most flexible yet popular model is the General Time Reversible (GTR) model.^23^ It assumes time reversibility and assigns distinct substitution parameters to the nucleotide pairs.^24^

**Fig. 1.**
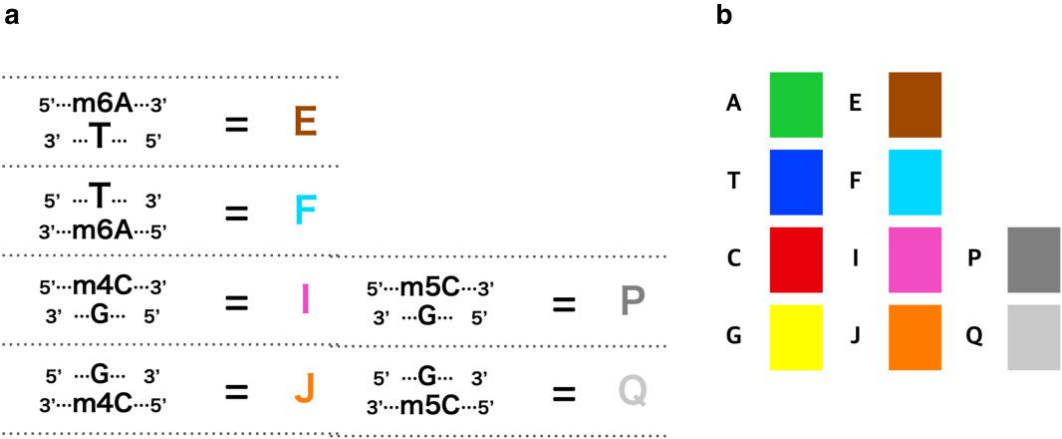
Definition of a 10-base system with methylated bases. (a) The one letter code for methylated bases. E: m6A of forward strand. F: m6A of reverse strand. I: m4C of forward strand. J: m4C of reverse strand. P: m5C of forward strand. Q: m5C of reverse strand. (b) The colours used to represent the 10 states (ordinary bases and methylated bases) are as follows. Some colours do not have formal names, so Hex colour codes are listed: A: #1AC938, T: #023EFF, C: #E8000B, G: #FFFF00, E: #9F4800, F: #00D7FF, I: #F14CC1, J: #FF7C00, P: #808080, Q: #C8C8C8.

To facilitate the analysis of bacterial methylome evolution, we attempted to expand the standard molecular evolution theory by treating unmethylated bases (A, T, G, C) and methylated bases (m4C, m6A, m5C) in an equivalent way. Figure 1 illustrates our notation. Note that this notation also reflects the methylation status of the opposite base; therefore, “T opposite A” and “T opposite m6A” are distinct. In this study, we aimed to estimate the substitution rate matrix for the extended base set from methylome sequence data generated by PacBio sequencers. Thus, we considered eight bases, incorporating m4C and m6A methylations, but excluded m5C methylation due to its lower detectability with PacBio sequencers. We extend the conventional 4 × 4 nucleotide substitution rate matrix to an 8 × 8 matrix that incorporates these methylated states within the framework of the GTR model.

As the methylome data sets, we used those of *Helicobacter pylori*. *H. pylori* is among the organisms with the highest number of DNA methyltransferases, with each strain carrying a unique set of approximately 10 DNA methyltransferases in its ∼1.6 Mb genome (see REBASE; https://tools.neb.com/genomes/index.php?page=H). In many *H. pylori* strains, the m4C and m6A methylomes were previously decoded by PacBio sequencers.^25–28^ Transcriptome analyses and gene knockout experiments combined with their methylomes demonstrated that DNA methyltransferases function as hubs in a gene expression regulatory network.^29^ Besides, *H. pylori* exhibits several remarkable characteristics that make it particularly interesting from an evolutionary genomics perspective.^30^  *H. pylori* exhibits high mutation rates and frequent and fine mutual homologous recombination.^31^ Nonetheless, it maintains a distinct population structure that reflects ancient human migration, as its transmission is rare and occurs primarily in childhood. Once established, *H. pylori* can persist in the host for decades and may contribute to various diseases including stomach (gastric) cancer.^32^

Here, we estimated the substitution rate matrix for the extended base set incorporating methylation status, based on alignments of methylome across ∼100 *H. pylori* strains.

## 2. Materials and methods

### 2.1. Expanded nucleotide code involving methylated bases

In molecular evolution models, the states of molecules, typically base pairs or amino acids, are represented by a single letter. To make molecular phylogenetic inferences using the methylome data, we defined new states representing methylated bases. In eukaryotes, DNA methylation occurs mainly at the 5-position carbon atom of cytosine (5-methylcytosine; m5C). In contrast, in prokaryotes, methylation also occurs at the N6-position of adenine (N6-methyladenine; m6A) and the N4-position of cytosine (N4-methylcytosine; m4C) frequently. Therefore, we need to introduce three new states to represent these three types of methylated bases (Fig. 1). Furthermore, we need to introduce three more states to represent the methylation of their complementary bases. These six new states are represented by letters E, F, I, J, P, and Q, as follows: “E” is m6A on the the strand of interest, “F” is thymine with m6A on the opposite strand, “I” is m4C on the strand of interest, and “J” is guanine with m4C on the opposite strand. “P’ is m5C on the strand of interest, and “Q” is guanine with m5C on the opposite strand (Fig. 1).

In this study, we used the PacBio SMRT sequencing method to identify methylated bases, where m5C could not be reliably identified. Therefore, hereafter we focus only on two types of methylations, m6A and m4C, represented with four letters “E”, “F”, ‘I”, and “J”. Using these newly defined states, we tried to expand a conventional molecular evolution model to include methylation and demethylation events.

### 2.2. Extended molecular evolution model

Substitution models are Markov models that describe changes over evolutionary time. These models describe evolutionary changes in DNA sequences and estimate evolutionary distances.^33^ One of the most selected models for estimating evolutionary phylogenies is the GTR model. Each element of the GTR rate matrix can be represented below.


$$Q_{ij}\;=\;x_{ij\;\;}\pi_{j}$$

$x_{ij}$ represents transition parameters from base *i* to base *j* and *π_j_* represents the equilibrium probability of each base, where *i* and *j* are different bases (*i*, *j* ∈ {T, C, A, G}, *i* ≠ *j*). It can simply be extended by adding other bases than T, C, A, G into this model. For example, in the 10-base model including 3 types of methylation, *i* and *j* are in {T, C, A, G, E, F, I, J, P, Q}. In this research the evolutional model was applied to 8 base data, so the states for *i* and *j* are in {T, C, A, G, E, F, I, J}. We call this model “GTR8”.

As traditional GTR has the time reversibility(*π_i_Q_ij_* = *π_j_Q_ji_*), the extended GTR including GTR8 also have the characteristics. Time reversibility means that the evolutionary process is stochastically equivalent regardless of whether viewed from the direction of time progress or from the opposite direction. It is known that all the eigenvalues of the rate matrix of a reversible Markov chain are real numbers. This allows us to use a stable and efficient algorithm for calculating the stochastic transition matrix.^34^

Here, the phylogenetic analysis software RAxML-NG was used to estimate these parameters.^35^ RAxML-NG allows GTRs to be constructed using any kind of characters. By using RAxML-NG with newly defined characters (E, F, I, J) and existing characters for base (T, C, A, G), parameters for branch length, substitution parameter, equilibrium probability, and site heterogeneity were estimated.

### 2.3. Conversion to 8-state notation

We used 122 methylome data of different strains of *Helicobacter pylori* generated by PacBio SMRT sequencing, among which 55 were published previously,^36,37^ 53 were newly sequenced and remaining 14 were obtained from the REBASE PacBio database^38^ (see Table S1 for details). For genome annotations, we referred to the MBGD database (version 2018-01).^39^

The entire genomes of 122 strains were sequenced by PacBio SMRT sequencing. The m6A and m4C methylation sites and consensus motifs were then determined for each strain using SMRT Analysis 2.3.0 RS_Modification_and_Motif_Analysis.1 with default parameters. Then, for motifs that were methylated at *r* % or more of the target sites, all target bases were considered to be ‘methylated’, and the 4-base sequence data was converted to 8-letter sequence data by incorporating methylation status. In addition, we considered only motifs that are conserved among at least *n* strains (*r* and *n* are parameters). The rationale behind these criteria is that each methylation site is assumed to be associated with a specific methylation motif recognized by a particular methyltransferase, whereas methylation sites not associated with a clear motif may represent erroneous assignments. A high probability of methylation at each target site can be important for evolutionary analysis, even if actual methylation status may vary depending on factors such as environment conditions. Here, we somewhat arbitrarily set the parameters to *r* = 70 and *n* = 24, but varying these parameters did not substantially affect the overall proportion of methylation sites (Table S2).

At the same time, for the above 122 strains, orthologues were identified using DomClust^40^ and DomRefine,^41^ and core genes were identified using CoreAligner.^42^ As a result, a total of 1,450 core genes were obtained. Multiple alignments were made for them using Clustal Omega^43^ and poorly aligned regions were removed by the tool trimAl.^44^ After these analyses were performed using sequence data in 4-base notation, the created alignment was converted to 8-base notation. Finally, in the sequences of each orthologous group, sequences that were considered to be split genes and fusion genes were removed.

### 2.4. Flow of molecular phylogenetic analysis

**Fig. 2.**
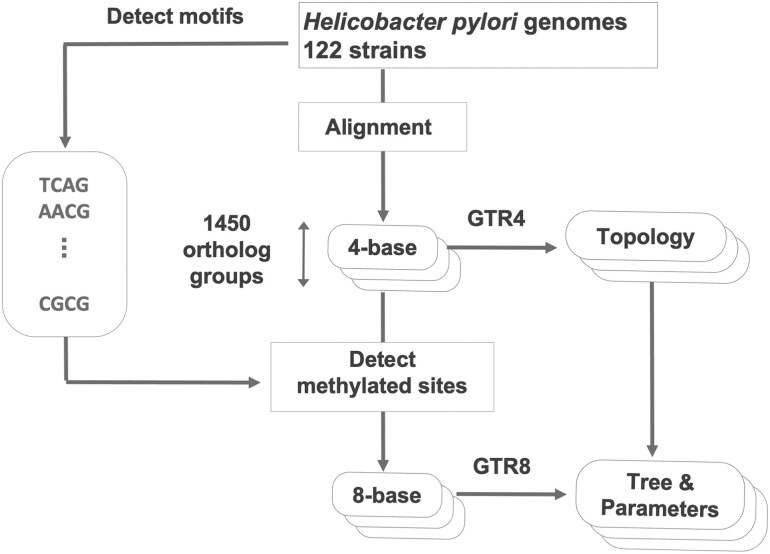
An overview of the entire analysis flow. We collected 4-base sequence data for 1450 orthologous groups by aligning and trimming raw sequence data from 122 *H. pylori* strains. In addition, we identified motifs from the raw sequence data. Using these motifs and the 4-base sequence data, we generated 8-base sequences for each orthologous group. The evolutionary tree topologies were estimated using the ordinary GTR model applied to the 4-base sequence. This process was repeated for all orthologous groups. Subsequently, the GTR8 model was applied to the 8-base sequence data to estimate the tree and evolutionary parameters such as rates and equilibrium parameters. In this process, the initial branch lengths were set to the values estimated by GTR4 and allowed to vary, while the topology was kept fixed based on the structure estimated by GTR4.

First, since each orthologous group is thought to have a different evolutionary background, we constructed a phylogenetic tree for each orthologous group using 4-nucleotide GTR (Fig. 2). Next, parameters of the GTR8 model were estimated by the maximum likelihood method while keeping the topology fixed to coincide with that of the GTR4 model. Branch lengths of the trees estimated by GTR4 model were regarded as initial values.

We employed GTR + G + I as the evolutionary model, in which the following parameters were estimated: branch lengths, 28 rate parameters, 8 equilibrium parameters, 4 shape parameters of discrete gamma, and one invariant site ratio. From the estimated parameters, we constructed a rate matrix for GTR8. At this time, in order to compare the parameters between different orthologous groups, we scaled them so that the sum of the flows, $\pi_{i}\;x_{i\;j}\;\pi_{j}$ (*i*≠*j*), equals one.


$$\overset{\;}{\underset{i\neq j}{\sum }}\;\overset{\;}{\underset{j}{\sum }}\;\pi_{i}\;x_{i\;j}\;\pi_{j}\;=\;1_{\;}^{\;}$$

Hereafter, we refer to this matrix ($\pi_{i}\;x_{i\;j}\;\pi_{j}$) as the normalized flow matrix.

## 3. Results

### 3.1. Substitution rate matrix and methylation dynamics

**Fig. 3.**
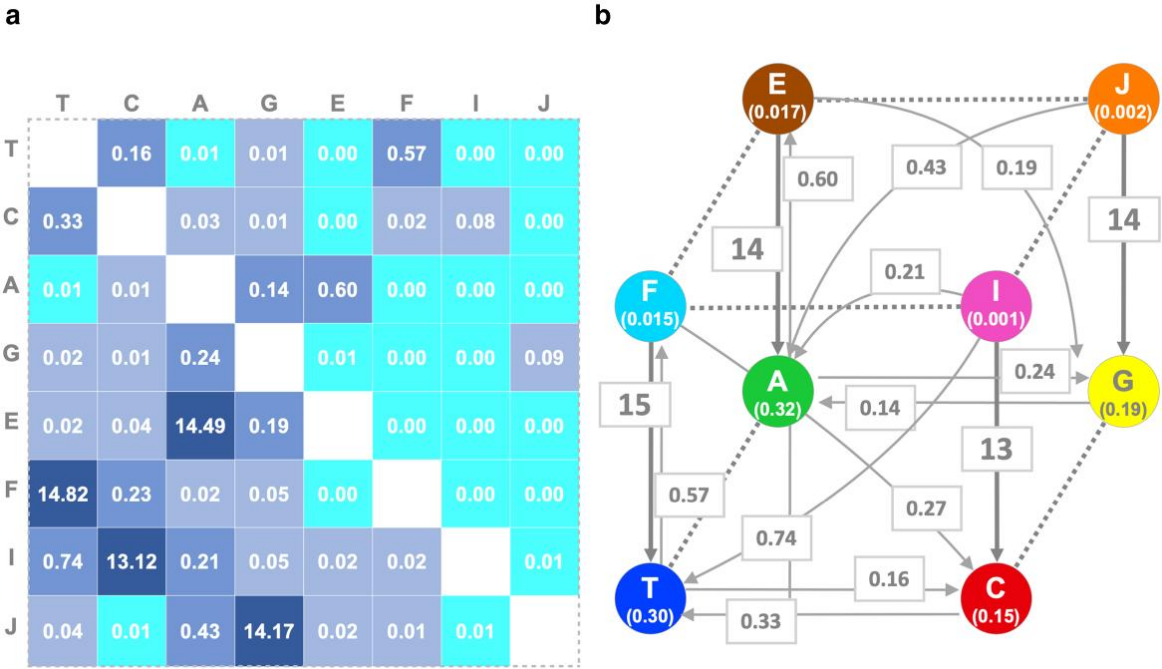
An overview of the estimated rate matrix and equilibrium parameters. (a) Averaged parameters of the estimated rate matrix over 1,450 ortholog groups. The maximum likelihood estimates of the rate parameters ($x_{1},\;\ldots ,\;x_{28}$) and equilibrium parameters($\pi_{A},\;\ldots ,\;\pi_{J}$) were obtained individually for each of the 1,450 ortholog groups. Then the rate matrices were constructed such that each element is represented as $x_{ij\;}\pi_{j}$ and scaled so that the sum of the non-diagonal elements is 1. Finally, each element of the scaled rate matrices was averaged. In the figure shown, each element is highlighted based on its magnitude. (b) Cubic visualization of base substitutions. Each vertex of the cube represents a base, with the average value of the equilibrium parameters shown in the parentheses. The bottom face of the cube represents the normal bases, while the top face represents the methylated bases and their complements. Each arrow represents the corresponding element of the rate matrix in (a). Arrows with values below 0.10 are omitted.

We tried to extend the GTR model^23^ to include the 8 states in a rate matrix with 56 elements ((8−1)×8). We applied the RAxML-NG program^35^ to the 8-base sequence alignments of 1,450 ortholog groups with 122 strains of *H. pylori* (see Materials and methods) and estimated the rate matrix for each ortholog group. The average values for the estimated matrix elements over all the ortholog groups are shown in Fig. 3a.

The highest substitution rates (shown in the darkest blue) were observed for demethylation events (E→A, F→T, I→C, J→G). The methylation rates (A→E, T→F, C→I, G→J) were about two orders of magnitude lower than the demethylation rates. Roughly speaking, these methylation rates were comparable to the substitution rates between the standard bases (A, C, G, T), but among them, the estimated substitution rates for transitions (A→G, G→A, C→T, T→C) were higher than those for transversions (A→C, A→T, C→A, C→G, G→C, G→T, T→A, T→G), consistent with existing knowledge. Substitution rates from a base to a different base with methylation (T→E, T→I, T→J, C→E, C→F, C→J, A→F, A→I, A→J, G→E, G→F, G→I) and from a methylated base to a differently methylated base (E→F, E→I, E→J, F→E, F→I, F→J, I→J, J→I) seem unlikely to occur intuitively. Indeed, these elements were estimated to be close to zero.

In Fig. 3b, we visualized the entire flow in a cubic network with the substitution rates and the equilibrium base frequencies. While the estimated equilibrium probabilities of methylated bases (E, F, I, J) are remarkably small ($\pi_{E}=0.017,$  $\pi_{F}=0.015,\pi_{I}=0.0013,\pi_{J}=0.0019$), the equilibrium probabilities of normal bases are higher by orders of magnitude ($\pi_{T}=0.30,\pi_{C}=0.15,\pi_{A}=0.32,\pi_{G}=0.19$). Those equilibrium probabilities are not far from the actual base composition (T = 0.27, C = 0.17, A = 0.31, G = 0.22, E = 0.015, F = 0.013, I = 0.0015, J = 0.0019). Given the remarkably high rate of demethylation, a methylated base is expected to be immediately converted back to an unmethylated base.

Our results also contain substitution rates from methylated bases to other bases (ie to bases other than their unmethylated form such as I → T or J → A). In general, the rates of these substitutions were similar to those of the corresponding substitutions between unmethylated bases (for the above example, C→T and G→A, respectively). We did not observe a large difference of base substitution triggered by methylation as suggested in m5C → T transition at CpG sites in mammalian genomes.^45^

### 3.2. Characterization of normalized flow matrices and orthologous groups

The above rate matrix can be reparameterized in the form of the state probabilities and the normalized flows in equilibrium. By definition of the GTR model, the normalized flows are represented as a symmetric matrix, which we refer to as the normalized flow matrix. Therefore, we can reduce the number of parameters by extracting only the upper triangular component.

**Fig. 4.**
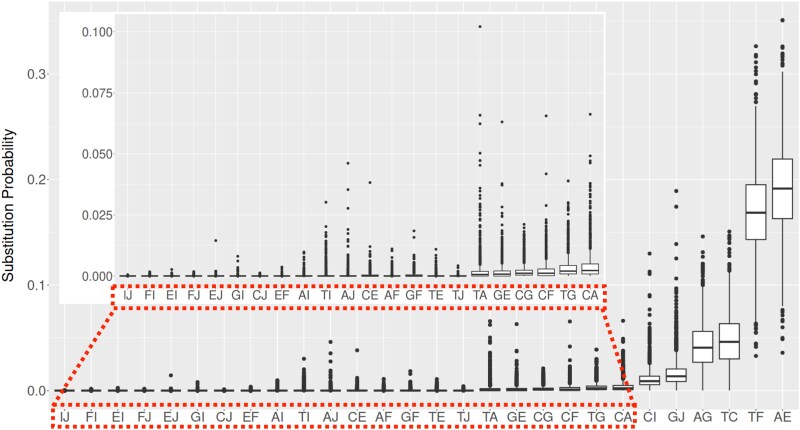
Box plot of estimated elements in the normalized flow matrix. A normalized flow matrix was created for each ortholog group by filling its elements with $\pi_{i}\;x_{ij\;}\pi_{j}$ and scaled so that the sum of the non-diagonal element is 1. Because of the symmetry of our extended GTR model, only the upper elements of the matrix are shown. With the exception of AE, TF, TC, AG, GJ, and CI, the estimated values are extremely small. Therefore, an enlarged plot of the remainder is displayed in the inset.

Figure 4 illustrates the box plot of the probabilities of substitution events obtained from the 1,450 ortholog groups. The most frequent substitutions were methylation/demethylation at m6A (AE and TF). The next were normal base substitutions classified as transitions (TC and AG), followed by m4C methylation/demethylation (GJ and CI), and then normal base substitutions classified as transversions. However, the probabilities of these substitutions varied depending on the ortholog groups as indicated by the whiskers. Substitution frequencies are much different between those involving m6A (E, F) and m4C (I, J) although there was no significant difference in their methylation rates in the substitution rate matrix. This is because the values of the equilibrium probability differed by about one order of magnitude between them (Fig. 3b).

### 3.3. Characteristic genes in terms of normalized flow

So far, our exploration has focused on interpreting the full spectrum of *H. pylori* genome evolution through the perspective of DNA base methylation/demethylation. Here, we focus on whether and how the spectrum of the substitution, including methylation/demethylation, varies among the 1,450 genes. For this purpose, we selected two major categories of mutation events: methylation/demethylation (AE, TF, CI, GJ) and base substitution (AG, TC, CA, TG, CG, TA). We then summed the probability values for each category, ranked them based on the total, and finally identified the top 10 ortholog groups (Table 1; see Table S3 for the complete table).

**Table 1.** Top 10 orthologous groups ranked by probabilities of mutation events in different categories: (A) methylation and demethylation (SUM METH) and (B) base substitution (SUM BASE SUB).

(A)			
Rank	Locus_Tag	Description	SUM METH
1	PS235		0.498
**2**	**HP1315**	**30S ribosomal protein S19**	**0.494**
3	HP0277	Ferredoxin	0.491
4	HP1242	Hypothetical protein (DUF465)	0.490
**5**	**HP1308**	**50S ribosomal protein L24**	**0.488**
6	HPF16_RS06545 (HPF16_1274)	Agmatine deiminase	0.486
**7**	**HP0126**	**50S ribosomal protein L20**	**0.485**
8	HP0386	Hypothetical protein (DUF3786)	0.485
9	HP1298	Translation initiation factor IF-1	0.483
**10**	**HP0491**	**50S ribosomal protein L28**	**0.483**

(B)			
Rank	Locus_Tag	Description	SUM BASE SUB
1	HPF16_RS03445	Hypothetical protein (HP0673/HP0674)	0.336
2	JHP_RS06750	Hypothetical protein	0.299
**3**	**HPF16_RS07265** **(HPF16_1417)**	**DNA methyltransferase (Type III RM)**	**0.269**
4	**HP1471**	**Type IIG RM system specificity protein** ^a^	**0.265**
5	HPF16_RS03805 (HPF16_0750)	VacA-like toxin, VacA-2 (HP0610)	0.265
6	HP0059	Hypothetical protein (long coiled coil)	0.265
7	HP1165	MFS transporter (multidrug transporter)	0.264
**8**	**HP0462**	**Type I RM system specificity protein**	**0.263**
9	ST1033	Bacterial Antiviral Defense Nuclease (HPG27_188)	0.262
10	HPF16_RS04485 (HPF16_0880)	(HP0897) DnaB modulator, DciA	0.262

Ribosomal protein genes (A) and genes in RM systems (B) are highlighted in bold.

^a^The annotation was taken from Furuta & Kobayashi.^46^

Remarkably, when ranked based on probabilities associated with methylation/demethylation, four of the top 10 ortholog groups are annotated as ribosomal protein genes (Table 1A). On the other hand, when ranked based on probabilities of base substitutions, three out of the top 10 ortholog groups are associated with RM systems. Ribosomal proteins are known as slowly evolving proteins at the base substitution level. In fact, the ascending order of methylation is nearly identical to the descending order of base substitution (see Table S3). This could be due to the mathematical constraints of the normalized flow matrix, where the sum of non-diagonal elements equals 1. In other words, the combined probability of both base substitutions and methylation/demethylation events tends to dominate the matrix, resulting in an inverse proportional relationship: (1 − (base substitution) ≒ (methylation/demethylation)).

**Fig. 5.**
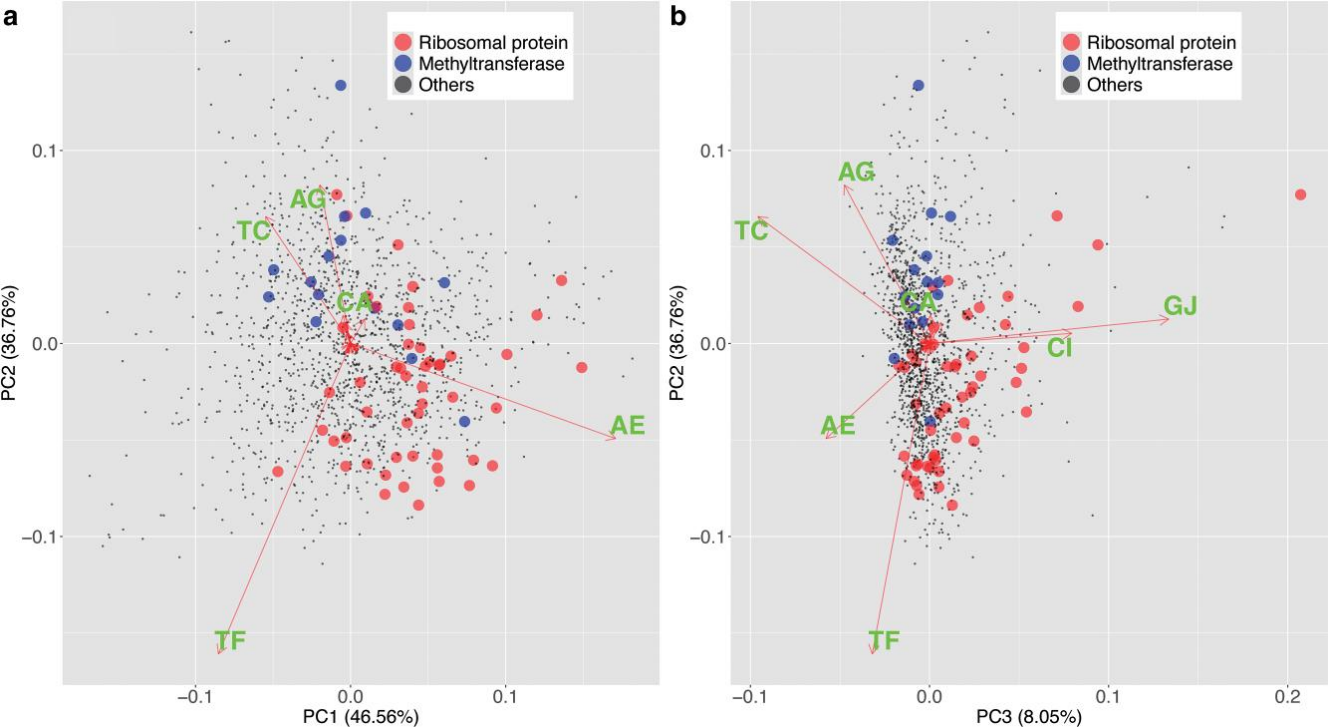
Principal component analysis (PCA) highlighting ribosomal protein genes (red) and DNA methyltransferase genes (blue). By rotating the figure (b) around the PC2 axis as the origin by 90 degrees and aligning it with the figure (a), the dispersion of the points in three dimensions can be evoked. The lengths of the arrows are scaled to prevent significant overshooting beyond the point cloud.

To visually validate these potential characteristics, we performed Principal Component Analysis (PCA) utilizing the probabilities of mutation events of the 1,450 ortholog groups. Subsequently, we constructed two-dimensional plots to represent the first three principal components (PCs), overlaying this plot with annotations of the two above mentioned groups, ribosomal proteins and DNA methyltransferases (Fig. 5). To account for the potential differences in estimation errors among substitution patterns that might affect the results of PCA, we also attempted to eliminate such effects using bootstrap analysis prior to PCA, but found no substantial differences in the outcomes (see Supplementary text).

Methylation and demethylation of m6A (AE and TF) with large variances roughly form the first principal component (explained variance ratio is 46.6%) and the second principal component (explained variance ratio is 36.8%), respectively (Fig. 5a). The result that AE and TF form distinct axes suggests that m6A methylation may exhibit asymmetry between the forward and reverse strands. This is consistent with the general asymmetry of the Type I and Type III R–M systems. On the other hand, in Fig. 5b, both methylation and demethylation of m4C (CI and GJ) are along the third principal component (explained variance ratio is 8.05%). This could be attributed to the influence of Type II RM systems with a palindromic recognition sequence. Cumulative variance of these three components exceeds 90%, indicating that they primarily account for the variance in substitution rates among different genes. Transitions (TC and AG) are oriented in the opposite direction to the sum of the methylation/demethylation vectors (AE, TF, and CI/GJ) along each principal component. The opposite directions between methylation/demethylation and substitution are again likely to be the consequence of the above mathematical constraint on the normalized flow matrix.

When 50 genes encoding ribosomal proteins were visualized on the PCA plot (Fig. 5), they were generally scattered in the direction of methylation/demethylation as opposed to base substitutions. This likely reflects the conservation of nucleotide sequences of ribosomal proteins within a species. Similarly, when 15 genes classified as DNA methyltransferases were visualized on a PCA plot, few of them were distributed in the positive direction of methylation (CI, GJ, TF). Instead, they appeared to be distributed in the positive direction of base substitutions (eg, TC, AG). This indicates that the diversity due to methylation is small, while the diversity due to base substitution is large, in contrast to the ribosomal proteins. Many bacterial DNA methyltransferases form a R–M system with a restriction enzyme, resulting in a high probability of self-cleavage by restriction enzymes when their methylation statuses change. This may partly explain the above observation, but it might also reflect the relatively low conservation among methyltransferase genes.

Ribosomal genes generally present in the positive direction of methylation across PC1, PC2, and PC3, yet there are genes that exhibit a negative direction of methylation specifically on PC2. Methyltransferases, on the other hand, are predominantly found in the negative direction of methylation on PC1 and PC2, while approximately zero on PC3, albeit there exist certain genes in the positive direction of methylation on PC1. We first expected that AE and TF are along the same principal component but they formed 2 different components, PC1 and PC2, respectively. The observed variance between PC1 (mainly AE) and PC2 (mainly TF) might result from non-palindromic methylation sites distributed asymmetrically in terms of the direction of transcription.

## 4. Discussion

In this research, we extended the existing model for the molecular evolution of nucleotide substitution to jointly analyse nucleotide substitution, base methylation, and demethylation. We applied this extended model to a dataset of *H. pylori* methylomes generated by PacBio sequencers. Although this study used an 8-base notation model that includes the methylation states of only m6A and m4C due to the limitations of the current technology, recent progress has led to the emergence of tools for accurate detection of m5C at CpG sites.^47^ Using such technology, our 10-base evolutionary code is applicable to methylome analysis in higher eukaryotes. A key advantage of the extended notation system is its compatibility with a wide range of present sequence analysis tools for methylome data, requiring minimal modifications. This is demonstrated by our successful estimation of a substitution matrix using existing software. We expect that this notation system will be widely used in various applications in methylome analysis in the future.

The parameters we estimated for the *H. pylori* methylome disclosed that the rate of demethylation is strikingly high, while the rate of methylation is approximately the same as that of base substitution. This indicates that modifications in methylation status can have much greater impact than base substitutions in a short time scale. This feature may facilitate rapid adaptation to environmental changes. This study, for the first time, shows this point quantitatively through extension of the conventional molecular evolutionary models. Although the exclusion of m5C methylation in this study may affect the overall estimation of the substitution rate matrix, the proportion of m5C-methylated bases has been estimated to be of comparable order of magnitude to those of m4C.^48^ Therefore, we believe that the same tendency would be observed even if m5C methylation were included.

In this study, we focused on protein-coding sequences that share a vast majority of the core genome across *H. pylori* strains. Through PCA combined with a procedure for eliminating the effect of estimation errors, we were able to characterize genes in terms of base substitution and methylation/demethylation. Specifically, we showed that ribosomal protein genes are more likely to undergo methylation than base substitutions, whereas DNA methylation enzyme genes tend to avoid methylation than base substitution. The latter phenomenon is particularly interesting as it can be regarded as a sort of immunity, potentially referred to as “methylation immunity” by DNA methyltransferase genes and/or their sequence-specificity determinant genes (Table 1B). Here, a DNA methyltransferase gene or its specificity determinant gene avoids being methylated by DNA methyltransferases. This is compatible with the behaviour of the R–M systems as selfish genetic elements such as viral genomes and transposons.^49,50^ However, we should bear in mind that these observations could be influenced by the mathematical constraints on the normalized flow matrix, where the sum of the non-diagonal elements must equal 1. Partitioning sequence data by codon positions, particularly focusing on third codon positions, may help mitigate the effect of strong selection acting on the protein-coding sequences.

The frequent and fine homologous recombination between *H. pylori* genomes may prevent accurate inference of phylogenetic tree topology, thereby reducing the reliability of parameter estimation. However, alterations of methylation status are generally so extensive that this reduction in reliability is unlikely to substantially affect the key observation noted above that modifications of methylation status have a much greater impact than base substitutions.

Our GTR8 model assumed time reversibility as well as context independence in methylome sequence evolution. Violation of the time reversibility has been reported,^51^ particularly in relation to the deamination process of m5C at CpG sites.^52,53^ Although, in this study, m5C methylations were excluded due to the limitation of PacBio sequencers, violations could also occur in other methylated bases. While some phylogenetic inference tools, such as BEAST^54^ and IQ-Tree,^55^ support time-irreversible nucleotide substitution models, they do not accommodate extended models with arbitrary character sets. To examine the possibility of irreversible substitutions influenced by base methylation, we need to develop an expanded irreversible model that accommodates expanded character sets, which is an important direction for future studies.

In this study, we assigned methylation bases based on the methylation motifs of DNA methyltransferases. There are three possible processes to alter the methylation status: (i) Gain/loss of a methylation motif through gain/loss of a DNA methyltransferase gene. (ii) Alteration of a methylation motif through changes in a sequence-specificity determinant gene (a DNA methyltransferase gene or a specificity subunit gene of a R–M system). (iii) Alteration of the sequence at a methylation site. In the first and second cases (in trans), methylation states of all the motif sites are altered simultaneously. This extremely large change is the characteristic feature of the methylation/demethylation events in comparison to the conventional base substitutions. This can be facilitated by the dynamic evolution of R–M systems through horizontal gene transfer and recombination involving target recognition domains.^46,56^ These processes indicate that these motif-based methylation/demethylation events are not independent between sites and may require more flexible evolutionary models that can accommodate site-specific likelihoods. Context-dependent evolutionary models have been successfully applied to estimate the substitution rates of m5C at CpG sites in mammals.^53^ Applying similar models to *k*-mer contexts around a methylation site could be a potential approach to this problem. However, estimating the large number of parameters remains a significant challenge.

In this study, we applied the 8-base GTR model to 122 methylomes of *H. pylori* to evaluate molecular evolutionary dynamics, with a primary focus on the frequency of methylation and demethylation. Part of the results obtained in this study may reflect the characteristics of *H. pylori*, which is rich in R–M systems. Therefore, the discussions regarding the rates of methylation and demethylation may not necessarily apply to other organisms. However, we hope that these results serve as an initial comparative dataset, providing a foundation for methylome evolutionary analyses as more methylome data and analysis techniques become available.

## Supplementary Material

dsaf025_Supplementary_Data

## Data Availability

The sequence data of *Helicobacter pylori* strains determined in this study have been deposited in the DDBJ with BioProject IDs ranging from PRJDB5838 to PRJDB5842 and PRJDB16300. Detailed information can be found in Table S1. The scripts for converting alignments to the extended nucleotide code and for performing evolutionary model analyses based on the extended nucleotide code are available at the GitHub repository (https://github.com/i-uchi/methylome-evolution/).
