# Supplementary material for: Towards molecular evolutionary epigenomics with an expanded nucleotide code involving methylated bases
Source: DNA Res. 2025 Sep 16;32(6):dsaf025. doi: 10.1093/dnares/dsaf025 (PMC12666383; doi:10.1093/dnares/dsaf025)
Supplement: dsaf025_Supplementary_Data [file dsaf025_supplementary_data.zip › SuppData_legend.docx]

**Legend of supplementary materials:**

**Supplementary text**

PCA after correction for estimation errors.

**Supplementary table S1**

Details of the methylome data from 122 *H. pylori* strains used in this study.

**Supplementary table S2**

Nucleotide components (in the 8 letter code) of methylomes generated under different parameter settings.

**Supplementary table S3**

Substitution probabilities in the normalized flow matrices of the 1450 ortholog groups.
