# Supplementary material for: Towards molecular evolutionary epigenomics with an expanded nucleotide code involving methylated bases
Source: DNA Res. 2025 Sep 16;32(6):dsaf025. doi: 10.1093/dnares/dsaf025 (PMC12666383; doi:10.1093/dnares/dsaf025)
Supplement: dsaf025_Supplementary_Data [file dsaf025_supplementary_data.zip › Supplementary_text.pdf]

Supplementary text

PCA without estimation error

To characterize each orthologous group in terms of substitution parameters, including methylation and demethylation, we performed principal component analysis (PCA) on the substitution parameters estimated for each orthologous group (as shown in Figure 5). In this supplementary analysis, we further removed the potential influence of estimation errors specific to each substitution pattern using a bootstrap-based correction (described below).

Below, we outline the procedure used to remove the influence of estimation errors. In general, the estimated values include estimation errors.

Let us consider two substitution parameters of an orthologous group, x and y. The estimated values are $x + e_x$ and $y + e_y$, where e_x and e_y are estimated errors. Assuming that a certain parameter and the error of the other parameters (e.g. x and e_y) are independent, the covariance of the estimated values including errors can be decomposed as follows.

$$cov(x + e_x, y + e_y) = cov(x, y) + cov(e_x, e_y)$$

In order to apply PCA to $cov(x, y)$, covariance of e_x and e_y were subtracted from the covariance of the estimated values. The covariance of e_x and e_y was obtained from the result of bootstrap analysis. Specifically, we carried out sampling of the sequences for each orthologous group 100 times. For each of these sampled sequences, we reiterated the procedure outlined in the previous chapter. We then derived a variance-covariance matrix from the estimation results of the 100 bootstrapped samples. The average value among the orthologous groups (here we used 1442 orthologous groups for which bootstrapping could be performed) was used as $cov(e_{x,}, e_y)$.

The results of PCA with this error adjustment is shown in Supplementary Fig. S1 below. Since the error-adjusted PCA and the simple PCA (Fig. 5) yielded very similar principal component scores and overall patterns, we preset only the simple PCA in the main text for simplicity.

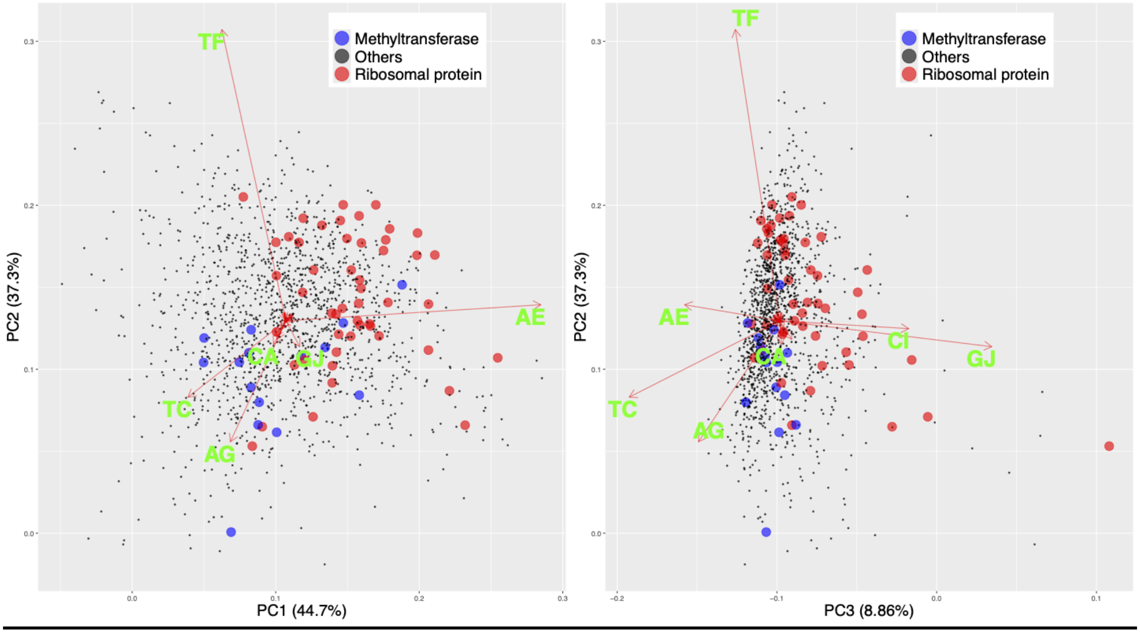

Supplementary Fig. S1 Principal component analysis after correction for estimation errors. The figure is drawn in the same way as Fig. 5. Note that the signs on the PC2 axis are reversed in comparison to Fig. 5.
